# TJP3 promotes T cell immunity escape and chemoresistance in breast cancer: a comprehensive analysis of anoikis-based prognosis prediction and drug sensitivity stratification

**DOI:** 10.18632/aging.205208

**Published:** 2023-11-10

**Authors:** Liu Chaojun, Li Pengping, Li Yanjun, Zhu Fangyuan, He Yaning, Shao Yingbo, Chen Qi, Liu Hui

**Affiliations:** 1 Department of Breast Surgery, Henan Provincial People’s Hospital; People’s Hospital of Zhengzhou University, People’s Hospital of Henan University, Zhengzhou, Henan 450003, China; 2 Center for Clinical Single-Cell Biomedicine, Henan Provincial People’s Hospital, People’s Hospital of Zhengzhou University, School of Clinical Medicine, Henan University, Zhengzhou, Henan 450003, China; 3 Breast Surgery, The First People’s Hospital of Xiaoshan District, Zhejiang, Hangzhou 311000, China

**Keywords:** anoikis-related genes (ARGs), breast cancer, machine learning, drug resistance, immunity

## Abstract

Background: Overcoming anoikis is a necessity during the metastasis and invasion of tumors. Recently, anoikis has been reported to be involved in tumor immunity and has been used to construct prognosis prediction models. However, the roles of anoikis in regulating tumor immunity and drug sensitivity in breast cancer are still not clear and therefore worth uncovering.

Methods: TCGA and GEO data are the source of gene expression profiles, which are used to identify anoikis-related-gene (ARG)-based subtypes. R4.2 is used for data analysis.

Results: Breast cancer is divided into three subgroups, amongst which shows prognosis differences in pan-cancer cohort, ACC, BLCA, BRCA, LUAD, MESO, PAAD, and SKCM. In breast cancer, it shows significant differences in clinical features, immune cell infiltration and drug sensitivity. Machine learning constructs prognosis prediction model, which is useful to perform chemotherapy sensitivity stratification. Following, TJP3 is identified and verified as the key ARG, up-regulation of which increases tolerance of paclitaxel-induced cell toxicity, accompanied with increased expression of caspas3 and cleaved-caspase3. In addition, Down-regulation of TJP3 weakens the cell migration, which accompanied with increased expression of E-cad and decreased expression of vimentin, twist1, zeb1, and MMP7. Furthermore, the expression level of PD-L1 is negative correlated with TJP3.

Conclusion: ARGs-based subgroup stratification is useful to recognize chemotherapy sensitive cohort, and also is useful to predict clinical outcome. TJP3 promotes chemoresistance, tumor metastasis and potential immunotherapy escape in breast cancer.

## INTRODUCTION

Tumor burden is a formidable disease for humanity and public health worldwide [1, 2]. As urbanization progresses, tumor incidence has varied from previous levels. According to Global Cancer Statistics 2020, breast cancer has the highest incidence in females [3]. Due to the anti-HER2 therapy regiment, HER2-positive breast cancer gets an obvious improvement in clinical outcome. However, malignant types of breast cancer, such as triple-negative breast cancer (TNBC), are still without powerful therapy strategy yet now, and 5-year survival rate is less than 30% [4].

Individualized treatment is prevalent for efficient anti-tumor therapy, and multi-gene-based assessment tools are widely applied in making adjuvant therapy strategies, such as 21-gene detection [5], genome detection and microsatellite instability detection [6]. Nowadays, diversity of pathway-based prognosis prediction model is explored and applied in identifying tumor subgroups, such as ferroptosis [7], cuproptosis [7], and immunogenic cell death [8] et al. Recently, anoikis has been reported to be involved in tumor immunity escape [9], and anoikis-related genes (ARGs) are applied to assess tumor immunity in glioblastoma [9] and neck squamous cell carcinoma [10]. However, it’s still unclear in breast cancer. Therefore, it is necessary to explore the roles of ARGs in regulating drug tolerance, tumor progression and prognosis prediction.

In this study, artificial intelligence is applied to construct ARG subtype stratification model, and we attempt to use AI to assess chemotherapy sensitivity in breast cancer. Finally, a key ARG is screened into further experiments.

## MATERIALS AND METHODS

**Figure 1:**
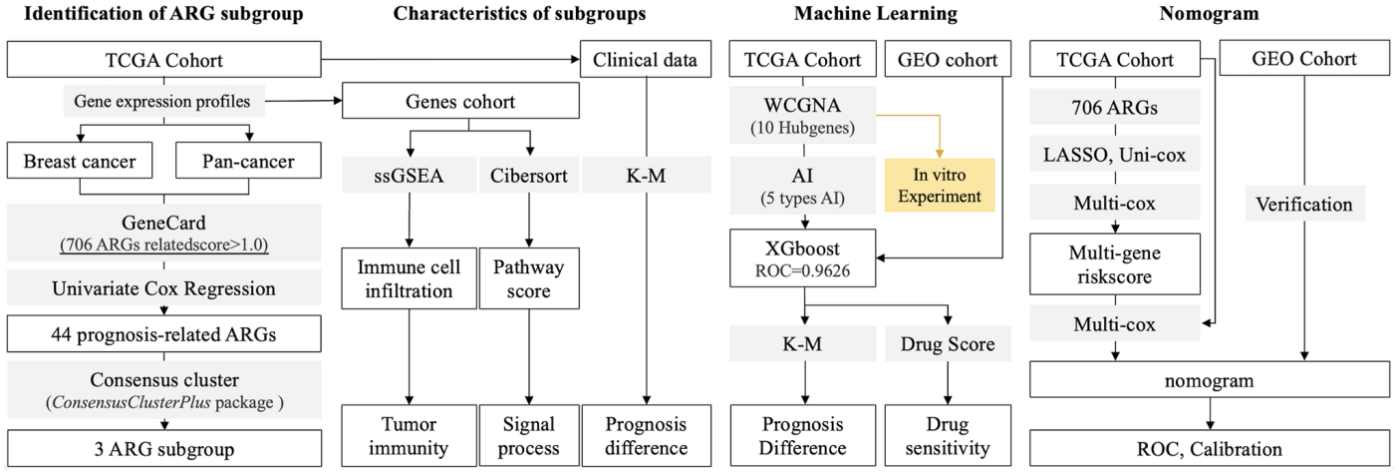
**Research Process.** (1) Identification of ARG subgroup: gene expression profiles are collected from TCGA and GEO databases, and ARG list is collected from GeneCard. 44 ARGs are selected into consensus cluster to identify subgroups. (2) Features in subgroups: Pathway score and immune cell infiltration are calculated by ssGSEA and CIBERSORT, respectively. (3) AI-based drug sensitivity stratification: 5 types of machine learning algorithms are applied, and XGBoost displays best results in identification of ARG subgroups. (4) TCGA (training) and GEO (testing) cohorts are used to construct prognosis prediction model. (5) Hubgenes are selected to perform *in vitro* experiments.

The aims of this research are to explore the roles of anoikis-related genes (ARGs) in identifying drug sensitivity subtypes (contains immunotherapy and chemotherapy), and to assess the roles of ARGs in predicting clinical outcome. The whole research network is shown in Figure 1.

### Bioinformatic analysis

#### Data collection

Data of gene expression is collected from The Cancer Genome Atlas (TCGA, https://portal.gdc.cancer.gov/repository) and Gene Expression Omnibus (GEO, https://www.ncbi.nlm.nih.gov/geo/). Clinical data is collected from Sangerbox [11], Kaplan Meier PlotteR (http://kmplot.com/analysis/index.php?), UALCAN (The University Alabama at Birmingham Cancer data analysis Portal, https://ualcan.path.uab.edu/tutorial.html), and GEO. TCGA cohort is used for model construction and training, while GSE25066 and GSE20685 are used for verification. GSE20685, GSE42568 and GSE58812 are used for breast cancer model verification, while GES30219, GSE231210, GSE37745, and GSE50081 are used for lung cancer model verification. Pan-Cancer Analysis of Whole Genomes (PCAWG, *n* = 818) data is used for pan-cancer model verification. Anoikis-related genes (ARGs) are collected from GeneCard (https://www.genecards.org). Immunohistochemical staining (IHC) of TJP3 is collected from The Human Protein Atlas (THPA, https://www.proteinatlas.org).

#### Prognosis analysis

Univariate Cox regression and Kaplan-Meier are used to calculate clinical outcome and hazard ratio (HR).

#### Subtypes identification and machine-learning-based drug sensitivity stratification

706 ARGs are collected from GeneCard, 44 of which are prognosis-related identified by Univariate Cox regression. 44 ARGs are put into consensus cluster analysis by R4.2.0 (*ConsensusClusterPlus*, *parameter: maxK = 10, reps = 50*). Then, identified subgroups are applied into supervised algorithm with genome (WGCNA) to find hub genes (module merging threshold is 0.3). Following, machine learning, containing Support Vector Machine (SVM, package: *e1071*), random forest (RF, package: *randomForest*), Extreme Gradient Boosting (XGboost, package: *xgboost*), multi-logistic (package: *nnet*), and deep learning (DL, package: *h2o*), is applied to construct training model (70% of TCGA cohort are training cohort, last 30%) to identify ARG subgroups in independent cohorts. Drug score is calculated by *OncoPredict* package in R4.2.0.

#### Tumor immunity assessment

Cibersort is used to predict immune cell infiltration in R4.2.0.

#### Pathway score

Genes related to ferroptosis (http://www.zhounan.org/ferrdb/current/), necroptosis (GeneCard), immunogenic cell death [12], cuproptosis (PMID: 35298263) [13], pyroptosis (GeneCard) and anoikis (GeneCard) are collected from corresponding databases or literatures. Simple sample Gene Set Enrichment Analysis (ssGSEA, package: *GSVA*) is applied to calculate aforesaid pathways score.

#### Drug score

Drug score is predicted by package *OncoPredict* in R4.2.0.

#### Nomogram

706 ARGs are put into Least Absolute Shrinkage and Selection Operator (LASSO) analysis, which is followed by Multivariate Cox regression. Five genes are finally selected to construct nomogram. Receptor operation curve (ROC) analysis and calibration analysis are applied to calculate the accuracy of the model, amongst which TCGA cohort is training cohort, while GEO cohort is testing cohort. Finally, we used nomogram to visualize the model.

### Biological experiments

#### Reagents

MBA-MD-231 is purchased from The Cell Bank of the Chinese Academy of Science in 2023 with STR matching analysis. Alive&dead staining kit is purchased from Yeasen Biotech, China, Edu staining kit is purchased from APExBIO, (K1077, USA). OPTI-MEM is purchased from (Thermo Fisher, Gibco, USA). Paclitaxel is purchased from CSNpharm (CSN19486, USA), and it is dissolved in DMSO. Antibodies against Caspase-3 (AF6311), cleaved-caspase-3 (AF7022, Affinity Biosciences, China), TJP3 (ab181991, Abcam, UK), PD-L1 (66248-1-Ig, Proteintech, China), E-cadherin (AF0131, Affinity Biosciences), Snail1 (AF6032, Affinity Biosciences), Twist1 (AF4009, Affinity Biosciences), Zeb1 (21544-1-AP, Proteintech), GAPDH (AF7021, Affinity Biosciences), MMP7 (10374-2-AP) are used for western blot.

#### Cell culture

The culture media of MBA-MD-231 is DMEM within 10% fetal calf serum and 100 units/mL penicillin and streptomycin.

#### Small interfering RNA (siRNA) experiments

Simply, triple-negative breast cancer (TNBC) cells are transplanted into 6 wells plates for 24 h, which is followed by transfection of TJP3 small interfere RNA (siRNA) (GenePharma, Shanghai, China) for 24 h, 48 h and 72 h. Transfection system: 250 ul OPTI-MEM + 6.5 ul siRNA (20 uM) + 13 ul Lipofectamine 3000 reagent (Invitrogen, USA). The siRNA sequences for TJP3 are listed in the following:

siRNA sequence of TJP3:

5′–3′ ACCUGCACCAAGAUGGCCAtt
3′–5′ UGGCCAUCUUGGUGCAGGUtt

#### Recombinant plasmid transfection assay

According to previous published work [14], the construction and transfection of recombinant plasmid are simply described as like the following: Primers of TJP3 is designed by Primer 5 soft, and the sequence is synthesized and inserted by PrimeScript RT Reagent Kit (TaKaRa, China), PrimeSTAR^®^ GXL DNA Polymerase (TaKaRa, China), SanPrep Column DNA Gel Extraction Kit (Sangon Biotech, China), and Hieff Clone™ Plus One Step Cloning Kit (Yeasen Biotech, China). For transfection, cells are transplanted into 6-well plate, and Hieff Trans™ Liposomal Transfection Reagent (Yeasen Biotech, China) is used to perform transfection. Transfection system: DMEM (10%FBS) + 2 ug plasmid + 4 ul transfection reagent. Finally, cells are harvested after 24~48 h.

#### Western blot

Cells with different treatments are harvested and lysed with RIPA lysis buffer (Sigma-Aldrich, USA), supplemented with phosphorylase and protease inhibitor mixture (Thermo Fisher Scientific, USA), quantified by the BCA assay (Beyotime, China). The whole process and protocol of western blot (WB) refer to our previous work (PMID: 31935687, 33282725).

#### Alive&dead staining

The alive&dead staining assay is performed with Calcein AM/PI staining assay (YEASEN Biotech Co., Ltd., China). Simply, cells are treated by different treatments, followed by co-culturing with Calcein AM and PI for 0.5~1 h. Then, the cells are washed by PBS for 2 times. Finally, cells are observed by fluorescent microscope (Green: alive cells; Red: dead cells).

#### Edu staining

Cells are transplanted into 24-well plates, followed by different treatments. Then, 5 ul Edu (20 uM) is added into cells for another 2 h. After that, cells are washed with PBS, and followed by 1% BSA culture for 1 h. Then, 0.25% triton-100 is used to penetrate cell membrane. Then, cells are with PBS and performed click reaction.

#### Transwell assay

Cells are transplanted into Transwell wells (24-well, 8.0 μm, Corning Incorporated, USA) with a 10% gradient of FBS for 48 h. Then stained by crystal violet for 15 mins. Quantification of passed cell area is performed by Image-ProR Plus.

### Statistics

All data analyses were performed in R4.2.0. Univariate Cox regression is performed to calculate the hazard ratio (HR) and the log-rank test is used to compare survival differences. Receiver operating characteristic (ROC) curves and the AUC value are performed by the *pROC* package in R4.2.0. *P* < 0.05 is considered to indicate a statistically significant difference.

### Data availability statement

The original contributions presented in the study are included in the Article/Supplementary Materials. Further inquiries can be directed to the corresponding authors.

## RESULTS

Conquering anoikis is necessary for epithelial-mesenchymal transition (EMT) [15], and the later one is considered as the key mechanism of tumor metastasis and invasion. Recently, anoikis has been reported to play roles in tumor immunity escape, and anoikis-related genes (ARGs) are also applied in constructing prognosis prediction tools. However, it is not comprehensively explored in breast cancer. This study aims to uncover the roles of ARGs in regulating chemoresistance and immunotherapy in triple-negative breast cancer (TNBC), and attempts to construct a ARGs-based multi-gene risk model, and to make a drug sensitivity prediction tool. The whole research designation is displayed in Figure 1.

### Identification of ARG-based tumor subtypes

**Figure 2:**
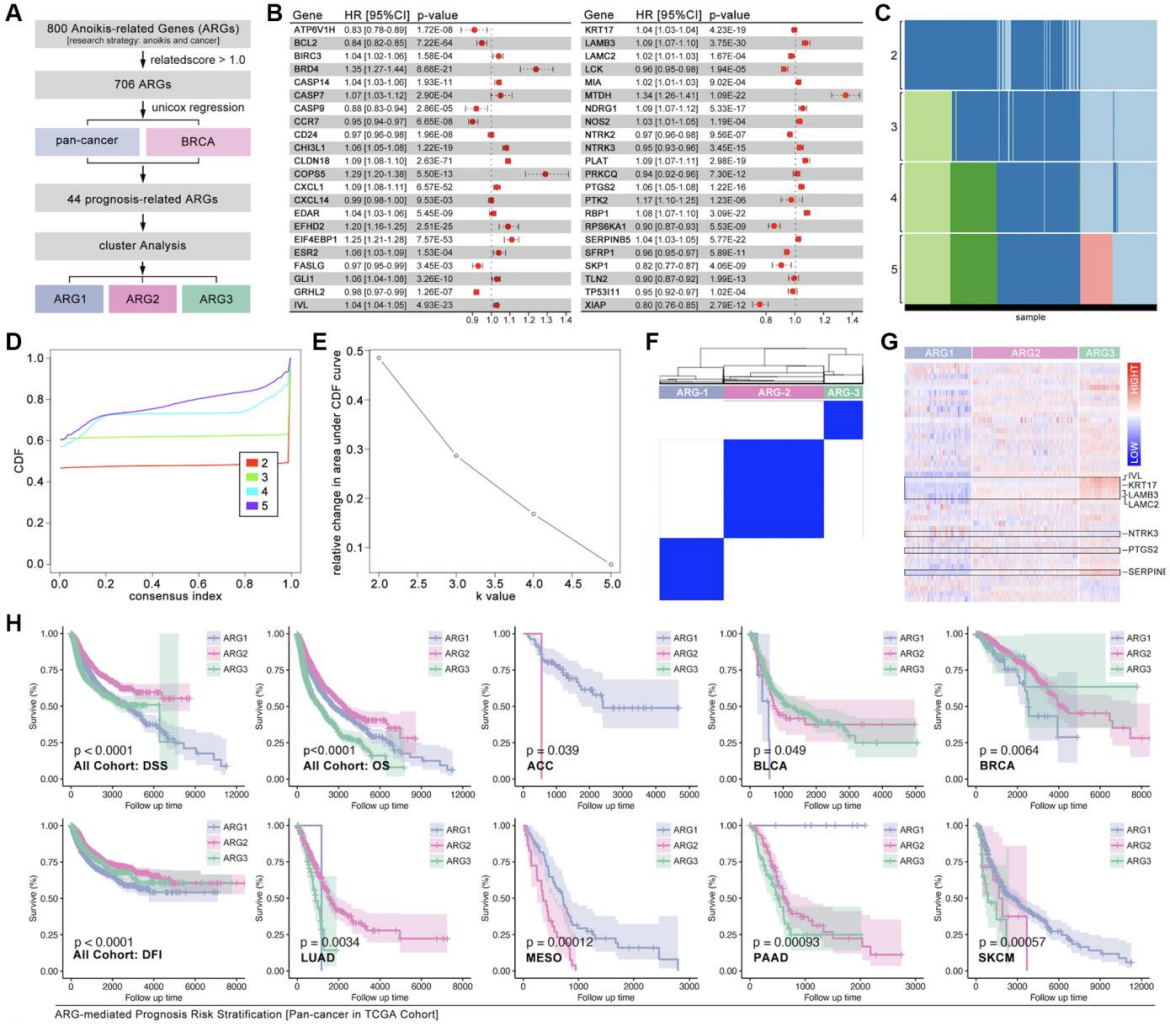
**Identification of ARG subgroups and prognosis features.** (**A**) ARG subgroups identification process. (**B**) The HR of 44 selected ARGs. (**C**–**F**) Consensus cluster analysis by *ConsensusClusterPlus* package to divide the TCGA cohort into three subgroups (ARG1, ARG2, ARG3). (**G**) 44 ARGs expression features amongst ARG subgroups. (**H**) Prognosis differences between ARG subgroups in pan-cancer and single type of cancer.

To redefine the tumor subtype, consensus cluster analysis is performed. Firstly, ARGs are collected from GeneCard with a research strategy of “tumor and anoikis” with a threshold related-score (>1.0) (Figure 2A). After Univariate Cox regression analysis, 44 ARGs are finally filtered out (Figure 2A). The hazard ratio (HR) of each selected ARGs is displayed, eighteen of which are protective factors, while twenty-six of which are risky factors in pan-cancer (Figure 2B). Following, the aforesaid 44 ARGs are put into consensus cluster analysis, and three-grouping strategy is the best strategy (Figure 2C–2F). The heatmap shows obvious differences of ARGs expression amongst ARG subtypes (Figure 2G). And the results show significant differences of overall survival (OS), disease-free survival (DFS) and disease-free interval (DFI) amongst ARG subtypes in pan-cancer cohort, while significant difference of OS is only observed in adrenocortical carcinoma (ACC), bladder urothelial carcinoma (BLCA), breast cancer (BRCA), lung adenocarcinoma (LUAD), mesothelioma (MESO), pancreatic adenocarcinoma (PAAD) and skin cutaneous melanoma (SKCM) (Figure 2H, *p* < 0.05).

### Features of tumor immunity and cell death signaling amongst ARG subtypes

**Figure 3:**
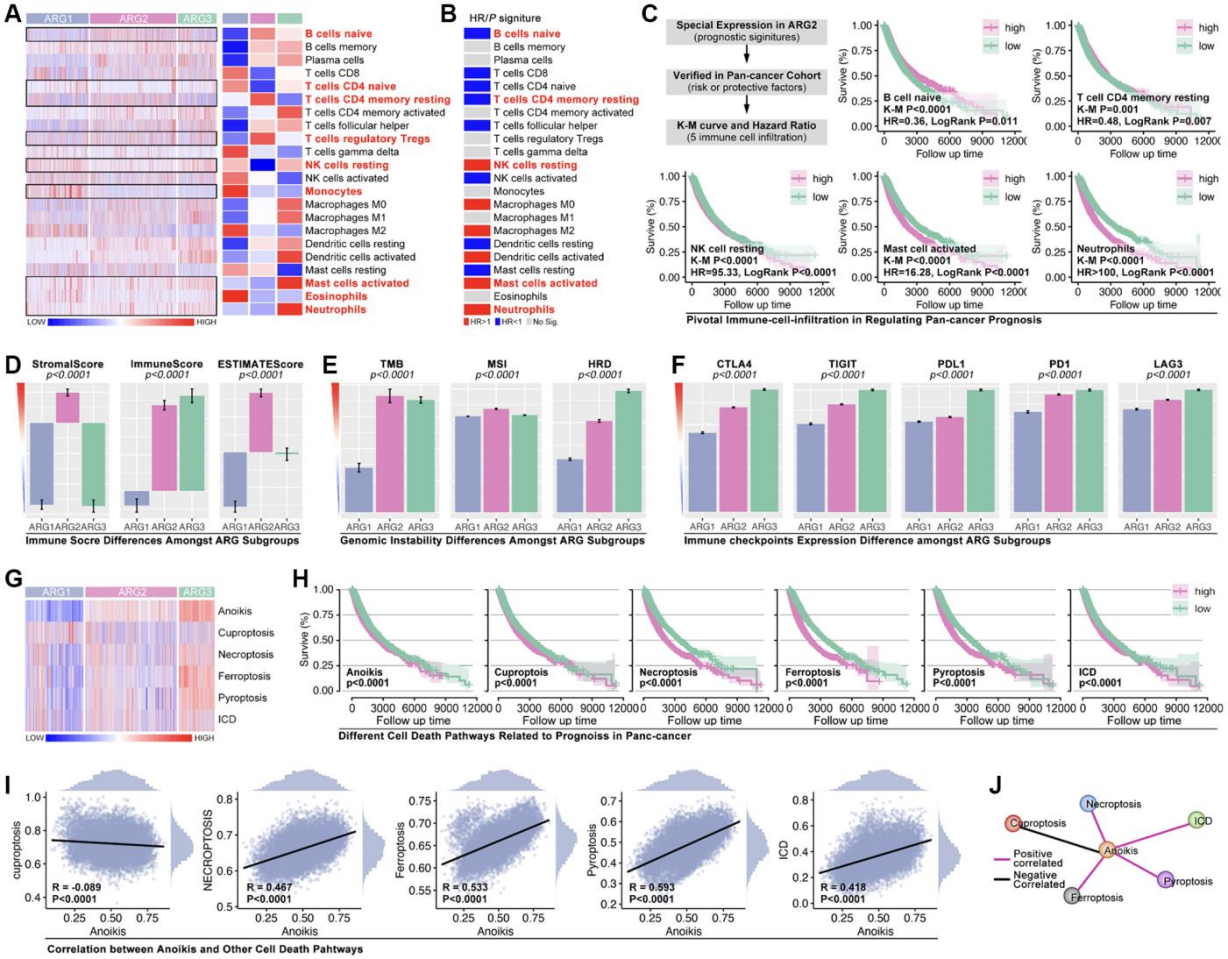
**Tumor immunity and pathway features amongst ARG subgroups.** (**A**) Cibersort is used to predict immune cell infiltration in pan-cancer. (**B**) HR of immune cells in pan-cancer. (**C**) Kaplan-Miler analysis and Univariate Cox regression of immune cells in pan-cancer. (**D**) Immune score is calculated by R4.2.0. (**E**) Genomic instability differences amongst ARG subgroups (data from Sangerbox: http://vip.sangerbox.com/home.html). (**F**) Immune checkpoints expression features in ARG subgroups. (**G**) Pathway score amongst ARG subgroups, calculated by ssGSEA. (**H**) K-M analysis of pathway score in regulating prognosis. (**I**, **J**) The relationship between anoikis score and the other pathway score.

Immune cells infiltration ratio analysis shows that significant difference exists in B cells naïve, T cells CD4 naïve, T cell CD4 memory resting, T cell regulatory (Tregs), natural killing (NK) cells resting, Monocytes, Mast cells activated, Eosinophils, and Neutrophils (Figure 3A, *p* < 0.05, labeled by red). Univariate Cox regression displays that B cells naïve, T cell CD4 memory resting, NK cells resting, Mast cells activated, and Neutrophils are prognosis-related factors in pan-cancer, in which forth three are protective factors while later four are risky factors (Figure 3B). Besides, results show that B cell naïve (K-M *p* < 0.0001, HR = 0.36, Logrank *p* = 0.011) and T cell CD4 memory resting (K-M *p* = 0.001, HR = 0.48, Logrank *p* = 0.007) are protective factors in pan-cancer, while NK cells resting (K-M *p* < 0.0001, HR = 95.33, Logrank *p* < 0.0001), Mast cells activated (K-M *p* < 0.0001, HR = 16.28, Logrank *p* < 0.0001), and Neutrophils (K-M *p* < 0.0001, HR > 100, Logrank *p* < 0.0001) are risky factors in pan-cancer (Figure 3C). Following, immune score is explored. As the results show, obvious significant differences of stromalscore, immunescore and estimatescore are observed amongst ARG subtypes (Figure 3D, *p* < 0.0001). And, genomic instability also holds a different trend amongst ARG subtypes as compared with immune score (Figure 3E). Furthermore, immunity checkpoint expression level amongst ARG subtypes is also uncovered. The results show that CTLA-4, TIGIT, PD-L1, PD-1 and LAG-3 holds same trend, which means ARG1 has lowest expression level of these genes while ARG3 has highest expression level of them (Figure 3F). In addition, cuproptosis, necroptosis, ferroptosis, pyroptosis and immunogenic cell death, are put into analysis. As Figure 3G shows, single sample GSEA (ssGSEA) analysis is applied to calculate pathway score, and the heatmap displays expression distribution in each sample and ARG subtypes (Figure 3G). Next, K-M analysis shows that all of foregoing pathways are risky factors in pan-cancer (Figure 3H), in which necroptosis, ferroptosis, pyroptosis and immunogenic cell death are positive correlated with anoikis, while cuproptosis is negative correlated with anoikis (Figure 3I, 3J).

### Machine learning redefining pan-cancer subtypes basing on anoikis

**Figure 4:**
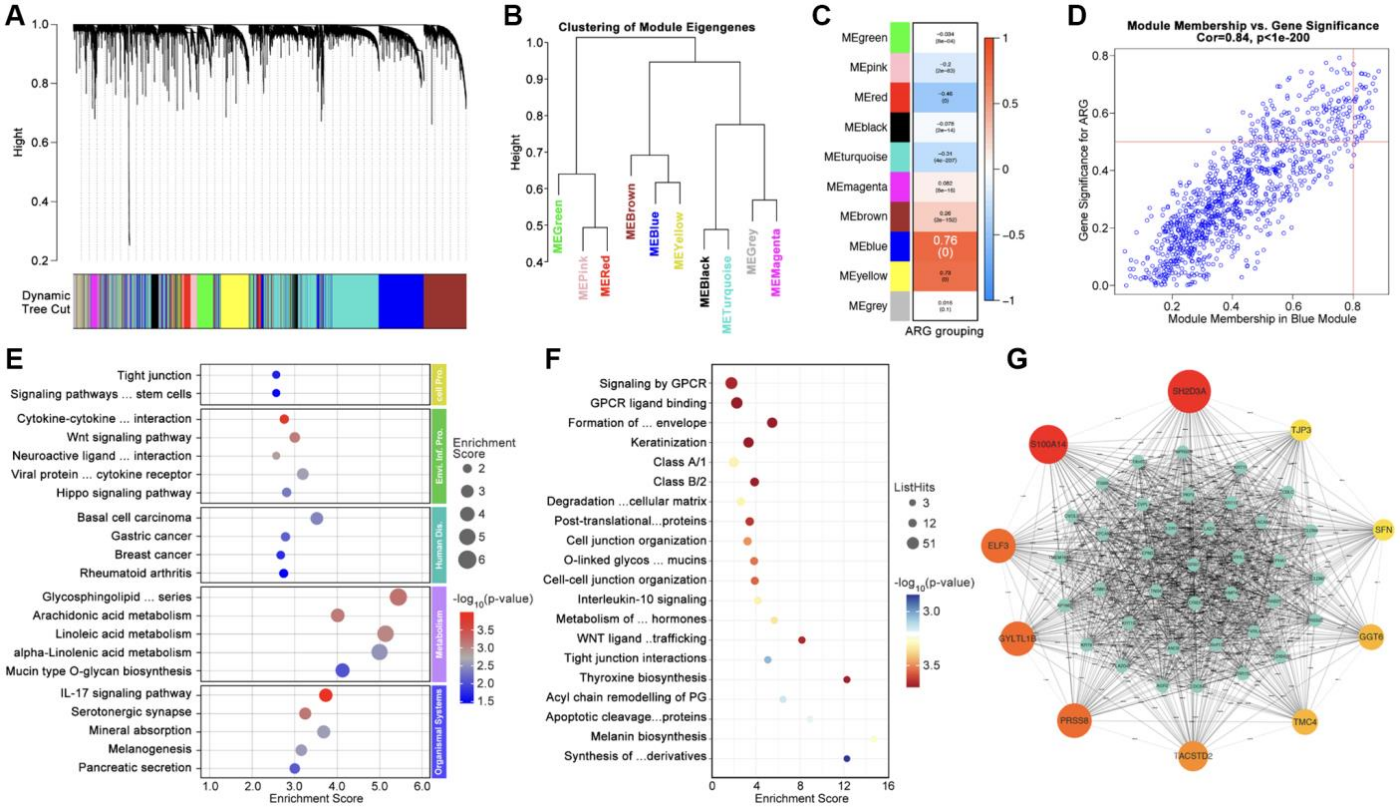
**Identification of Hubgenes amongst ARG subgroups.** (**A**, **B**) Dynamic tree cut and modules stratification of WCGNA. (**C**) Correlation between modules and ARG grouping. (**D**) The correlation between Module-blue and ARG grouping. (**E**) GO analysis and (**F**) KEGG analysis. (**G**) Interaction network of top 50 Hubgenes.

Firstly, WCGNA analysis is applied to identify hub-genes. As Figure 4A, 4B show, genome is divided into 10 subgroups with different colors (Figure 4A, 4B), amongst which Module blue (MEblue) holds the highest correlation between gene expression and ARG subtype identification (Figure 4C, 4D). Following, top-50 hub-gene are put into ARG grouping and GO/KEGG analysis, and results show that hub-gene is related to cell adhering, immunity and tumor process, et al. (Figure 4E, 4F). CytoScape recognizes the most important genes (SH2D3A, TJP3, SFN, GGT6, TMC4, TACSTD2, PRSS8, GYLTL1B, ELF3, and S100A14) for further analysis (Figure 4G).

**Figure 5:**
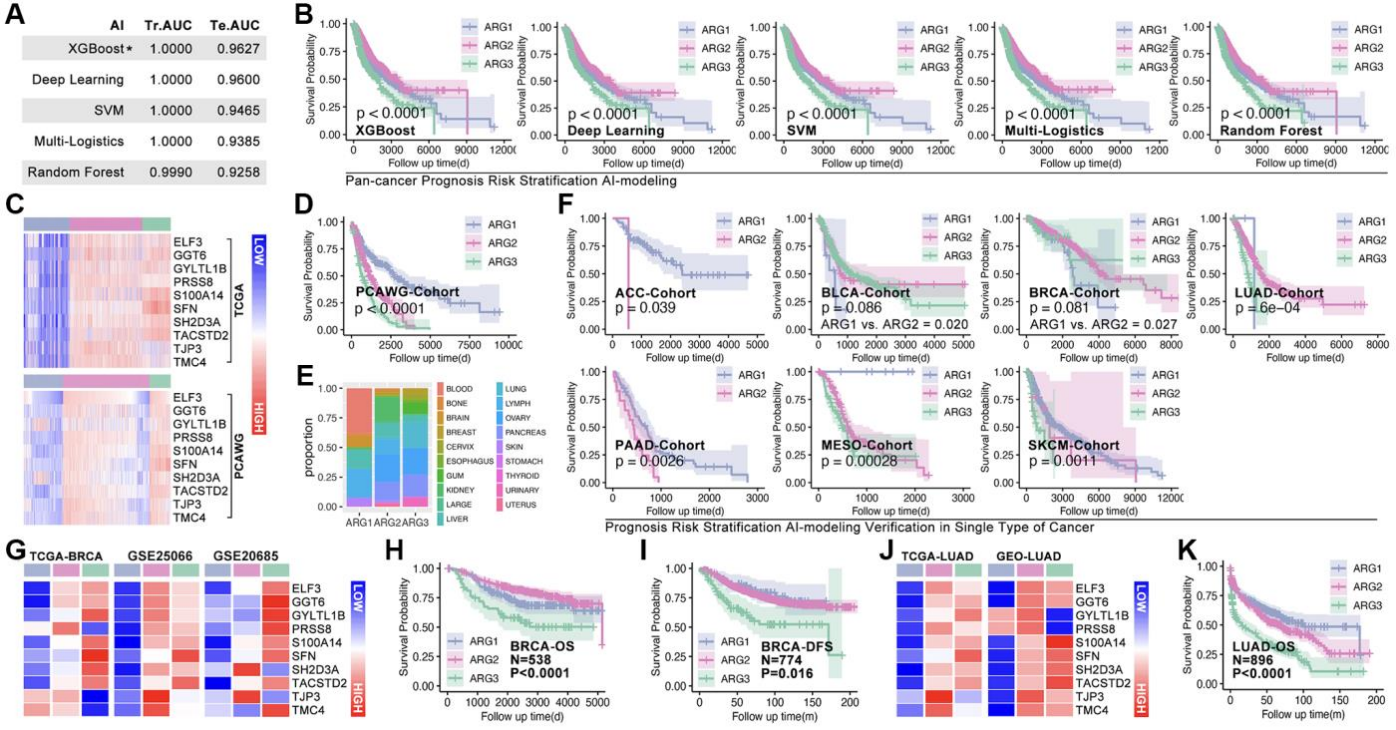
**Machine learning identifies ARG subgroup.** (**A**) TCGA cohort is divided into testing cohort (30%) and training cohort (70%), five types of machine learning algorithms are applied, and ROC (Tr.AUC means training AUC; Te.AUC means testing AUC) is used to assess the accuracy of models. (**B**) K-M analysis displays prognosis features amongst ARG subgroups. (**C**) Expression feature of Hubgenes in TCGA cohort and PCAWG cohort. (**D**) K-M analysis displays prognosis features amongst ARG subgroups in PCAWG cohort. (**E**) Pan-cancer distribution in ARG subgroups in PCAWG cohort. (**F**) AI-based ARG identification model recognizes ARG subgroups in single cancer in TCGA cohort, and K-M analysis shows prognosis features amongst ARG subgroups. (**G**) Expression feature of Hubgenes in breast cancer, whose data from TCGA and GEO cohorts. (**H**) Overall survival (OS) differences amongst ARG subgroups in GEO cohort (GSE20685, GSE42568). (**I**) Disease free survival (DFS) differences amongst ARG subgroups in GEO cohort (GSE21653, GSE25066). (**J**) Expression feature of Hubgenes in LUAD, whose data from TCGA and GEO cohorts. (**K**) OS differences amongst ARG subgroups in GEO cohort (GES30219, GSE231210, GSE37745, GSE50081).

Hubgene-based artificial intelligence (AI) is used to construct ARG subtype stratification model with TCGA cohort. Five types of algorithms show relatively good performance, and the XGboost is the best one, in which training AUC is 1.0 and testing AUC is 0.9627 (Figure 5A). Meantime, the prognosis is significantly different amongst ARG subgroups in pan-cancer (Figure 5B). To further verify the liability of model, independent cohort is applied. As Figure 5C shows, expression of hub-gene is obviously different amongst AI-identified ARG subtypes in PCAWG cohort (Figure 5C), and the ARG subtypes’ prognosis is also significantly different (*p* < 0.0001, Figure 5D). Tumor types distribution in ARG subgroups is displayed in Figure 5E. In order to explore whether the model is also workable in single type of cancer, 33 single types of cancer are put into analysis. Here, prognosis differences occur in ACC, BLCA, BRCA, LUAD, PAAD, MESO, and SKCM (Figure 5F). For further verification, we randomly selected breast cancer and lung cancer into independent cohort testing. First, expression features of hub-gene in TCGA cohort (BRCA) and GEO cohort (GSE20685, GSE42568 and GSE58812) are explored (Figure 5G), and K-M analysis shows differences of OS and DFS amongst subgroups in GEO cohorts (Figure 5H, 5I). Same analysis is performed in long cancer (Figure 5J, 5K).

### AI recognizes hierarchical chemotherapy sensitivity subtypes in breast cancer

**Figure 6:**
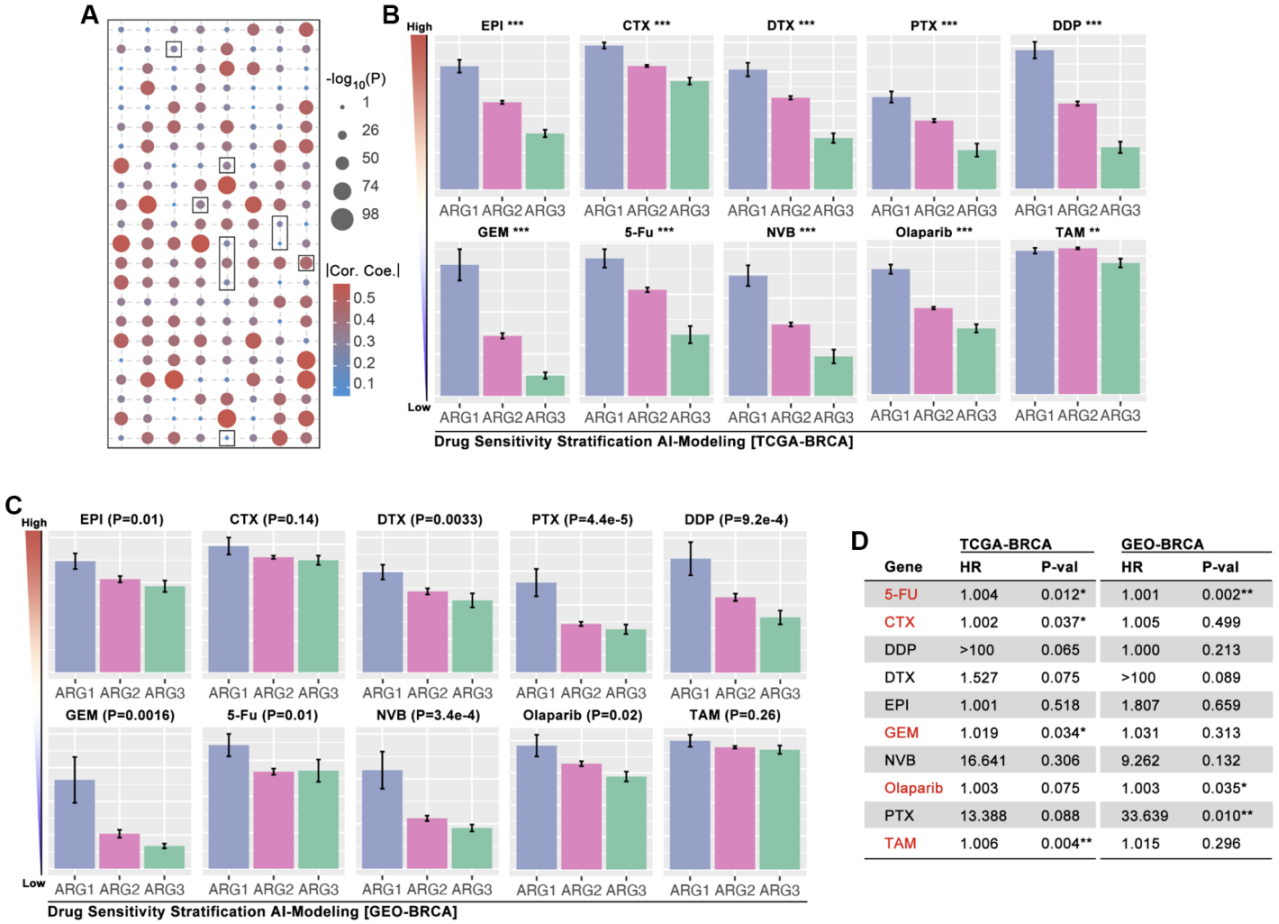
**AI-based drug sensitivity stratification.** (**A**) Correlation between drug score and anoikis pathway score, in which selected one are EPI, CTX, DTX, PTX, DDP, GEM, 5-Fu, NVB, Olaparib, and TAM (data from TCGA). (**B**) Drug score differences amongst ARG subgroups of BRCA (^*^ means *p* < 0.05, ^**^ means *p* < 0.01, ^***^ means *p* < 0.001, data from TCGA). (**C**) Drug score differences amongst ARG subgroups of BRCA (data from GES20685, GSE42568, GSE58812). (**D**) HR of drug scores in TCGA cohort and GEO cohort of BRCA.

In order to explore whether ARG-based model can identify drug sensitivity, OncoPredict algorithm is applied to calculate drug score. As Figure 6A shows that EPI, CTX, DTX, PTX, DDP, GEM, 5-Fu, NVB. Olaparib and TAM are selected, and which display significant difference amongst ARG subgroups (Figure 6B). Then, GEO cohorts (GSE20685, GSE42568, GSE58812) are applied to verify the drug stratification. As results show, same significant trends are observed in EPI, DTX, PTX, DDP, GEM, 5-Fu, NVB, and Olaparib (*p* < 0.05, Figure 6C), while no statistical significance exists in CTX and TAM (Figure 6C). Then, the role of drug score in breast cancer prognosis is explored, and it shows that 5-Fu, CTX, GEM, Olaparib and TAM are statistically significant risk factors (Figure 6D). Furthermore, the most sensitive drugs are also filtered, and Taselisib, Doramapimod, AZD8186, Ibrutinib and ZM447439 are the top5 drugs (Supplementary Figure 1).

### TJP3 promotes drug resistance and tumor metastasis in TNBC

**Figure 7:**
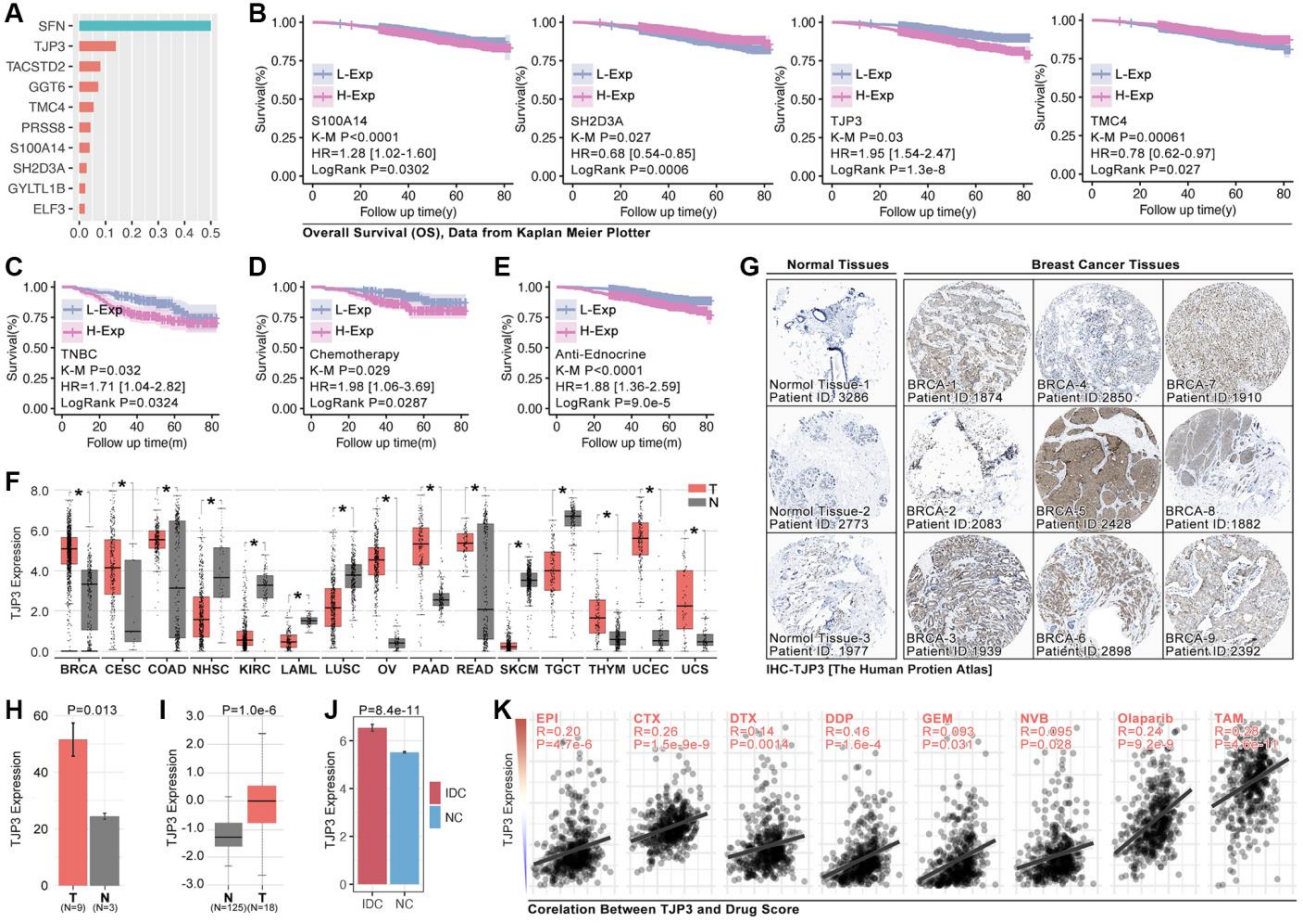
**TJP3 is a pivotal hubgene in regulating drug tolerance.** (**A**) Importance analysis in XGboost, in which SFN, TJP3, TACSTD2, GGT6, TMC4, PRSS8, S100A14, SH2D3A, GYLTL1B, and ELF3. (**B**) K-M analysis of S100A14, SH2D3A, TJP3, and TMC4 in regulating prognosis. (**C**) The roles of TJP3 in regulating prognosis in TNBC cohort, (**D**) chemotherapy cohort, and (**E**) endocrinotherapy cohort. (**F**) TJP3 expression in pan-cancer and adjacent tissues. (**G**) The expression level of TJP3 in adjacent tissues and breast cancer tissues (data from THPA). (**H**) Statistical results of TJP3 expression in THPA cohort. (**I**) TJP3 expression level in TNBC and corresponding adjacent tissues. (**J**) TJP3 expression level in IDC and corresponding adjacent tissues, data from GSE10780. (**K**) The correlation between the expression of TJP3 and drug scores (EPI, CTX, DTX, PTX, DDP, GEM, 5-Fu, NVB, Olaparib, TAM). ^*^p < 0.05, ^**^p < 0.01, ^***^p < 0.001.

To assess the roles of anoikis in regulating drug resistance and prognosis in breast cancer, an important analysis is performed. It ranks the importance of hub-gene in ARG subgroups identification, and the top3 genes are SFN, TJP3 and TACSTD2 (Figure 7A), amongst while only TJP3 (HR = 1.95, *p* = 1.3e-8) is prognosis-related gene in breast cancer (Figure 7B). Therefore, TJP3 is finally selected for further analysis. Results show that higher expression level of TJP3 are accompanied with worse clinical outcome in TNBC cohort, chemotherapy treatment, and endocrine therapy (data from Kaplan Meier PlotteR, Figure 7C–7E). In addition, RNA expression of TJP3 is higher in BRCA, CESC, COAD, OV, PAAD, READ, THYM, UCEC and UCS when compared with adjacent tissues (Figure 7F). IHC of TJP3 is collected from The Human Protein Atlas (THPA) and the results show that BRCA tissues hold higher expression level of TJP3 as compared with adjacent normal tissues (Figure 7G, 7H), same result is observed in UALCAN (The University Alabama at Birmingham Cancer data analysis Portal) breast cohort (Figure 7I) and GEO cohort (GSE10780) (Figure 7J). Finally, the correlation between TJP3 expression and drug score is explored, and it displays that TJP3 level is positive correlated with EPI (*r* = 0.20, *p* = 4.7e-6), CTX (*r* = 0.26, *p* = 1.5e-9), DTX (*r* = 0.14, *p* = 0.0014), DDP (0.16, *p* = 1.6e-4), GEM (*r* = 0.093, *p* = 0.031), NVB (0.095, *p* = 0.028), Olaparib (*r* = 0.24, *p* = 0.028) and TAM (*r* = 0.28, *p* = 4.6e-11) (Figure 7K).

**Figure 8:**
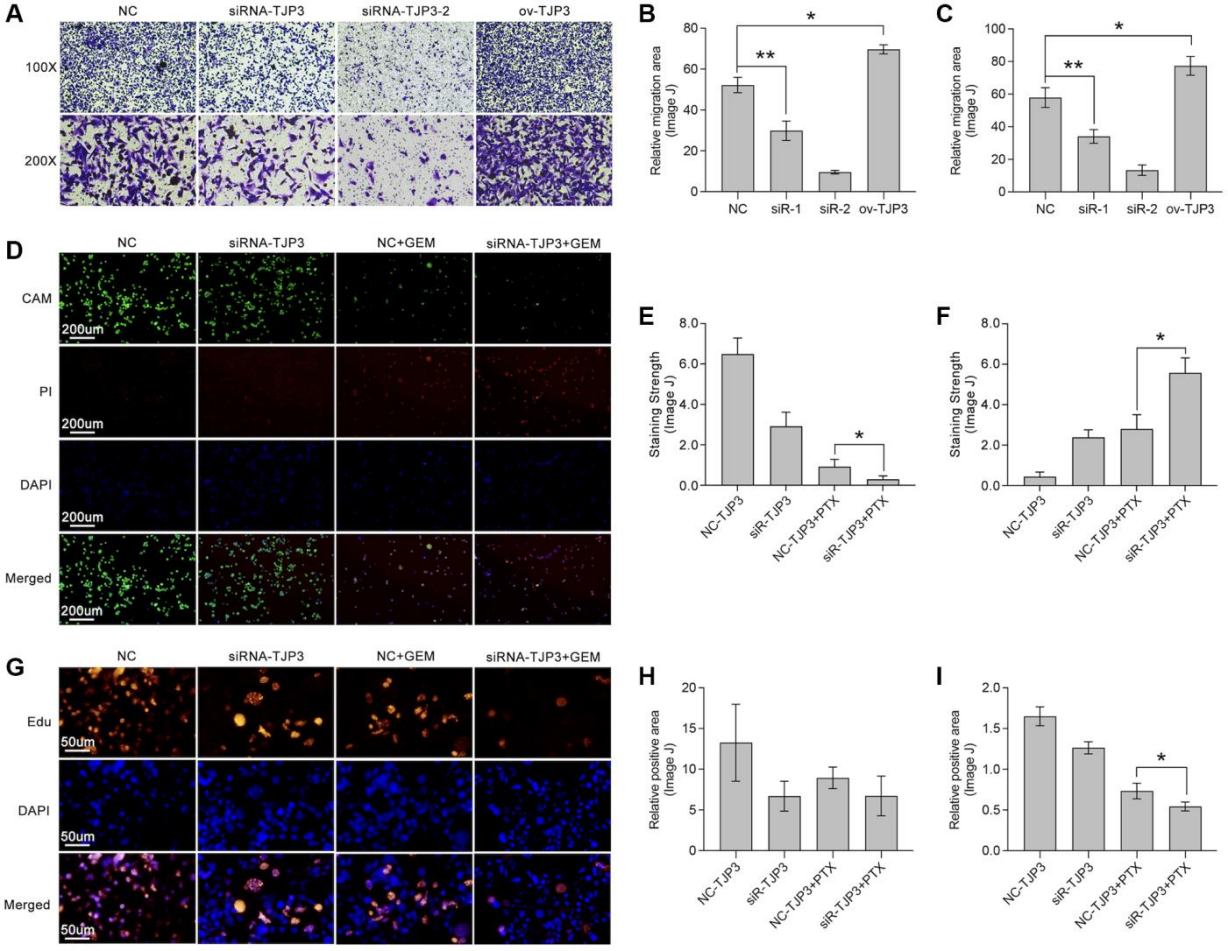
**TJP3 regulates migration and drug tolerance in TNBC.** (**A**) Transwell assay displays the role TJP3 in regulating cell migration. Statistical analysis shows migrated cell area in (**B**) 100-fold and (**C**) 200-fold field of view by ImageJ. (**D**) Alive&dead assay, in which (**E**) CAM means alive cells with green and (**F**) PI means dead cells with red. (**G**) Edu assay shows the role of TJP3 in regulating cell proliferation in (**H**) 100-fold and (**I**) 200-fold field of view by ImageJ. ^*^p < 0.05, ^**^p < 0.01, ^***^p < 0.001.

**Figure 9:**
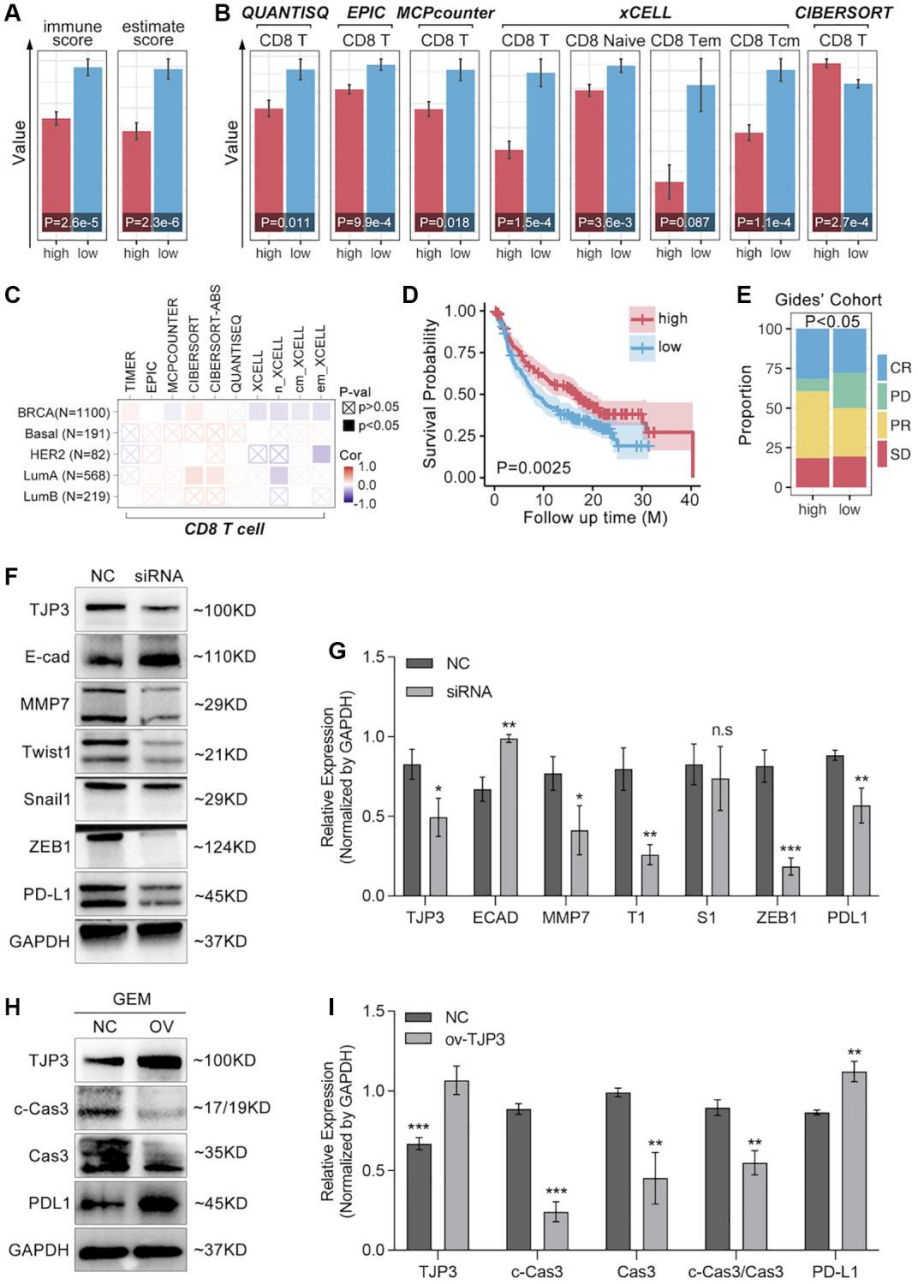
**TJP3 regulates GEM-induced cell apoptosis and EMT process.** (**A**) Immune Score predicted by SangerBox (http://vip.sangerbox.com/login.html). (**B**) T cell infiltration predicted by SangerBox (http://vip.sangerbox.com/login.html). (**C**) Immune cell infiltration predicted by TIMER2.0 (http://timer.comp-genomics.org/timer/). (**D**) Anti-PD1 prognosis in TJP3 low and high expression groups. (**E**) Anti-PD1 response differences between TJP3 low and high expression groups. (**F**) Si-RNA down-regulates TJP3 expression, and the corresponding down-stream targets expression features. (**G**) Statistical analysis, normalized by GAPDH. (**H**) Recombinant plasmid up-regulation TJP3 expression, and the corresponding down-stream targets expression features. (**I**) Statistical analysis, normalized by GAPDH. ^*^p < 0.05, ^**^p < 0.01, ^***^p < 0.001.

To further verify the roles of TJP3 in regulating tumor metastasis and drug tolerance, *in vitro* experiments are explored. siRNA is used to down-regulate the expression of TJP3 and recombinant plasmid is used to up-regulated TJP3 expression. As results show, down-regulation of TJP3 inhibits the migrated cells area about 30%-decreasing, as compared with control group (Figure 8A–8C), while over-expression of TJP3 promotes cell migration about 20%-increasing (Figure 8A–8C). Meantime, Alive&dead assay is performed, and the results show that down-regulation of TJP3 enhances the toxicity of GEM in breast cancer cells (*p* < 0.05, Figure 8D–8F). Furthermore, down-regulation of TJP3 enhances GEM-induced cell toxicity about 20%-increasing (*p* < 0.05, Figure 8G–8I). Based on the above results, epithelial-mesenchymal transition (EMT) and cell apoptosis are explored after changing the cellular expression of TJP3. As results show, siRNA down-regulates TJP3 expression about a 40% decreasing (*p* < 0.05), which leads up-regulation of E-cad (a 30% increasing, *p* < 0.01) and down-regulation of MMP7 (a 50% decreasing, *p* < 0.05), Twist1 (a 70% decreasing, *p* < 0.01) and Zeb1 (a 75% decreasing, *p* < 0.001) (Figure 9F, 9G). On contrary, over-expression of TJP3 (*p* < 0.001) leads down-regulation of cleaved-caspase3 and the ratio of cleaved-caspase3/caspase3 (more than a 50% decreasing, *p* < 0.01) (Figure 9H, 9I).

### TJP3 is a T cell immunity regulator for breast cancer

To further explore the relationship between TJP3 and T cell immunity, we apply bulky data analysis. As the Figure 9A shows, high-expression of TJP3 predicts lower immunescore and estimatescore (*p* < 0.05, Figure 9A), and the CD8 T cells infiltration is also lower in TJP3 high-expression group (Figure 9B). However, the correlation between the TJP3 and CD8 T cell infiltration is not definitive predicted by TIMER2.0 (Figure 9C). In order to uncover the underlying roles of TJP3 in regulating T cell immunity, we apply clinical trial cohort. As the Figure 9D, 9E show, higher expression of TJP3 predicts better prognosis in anti-PD1 therapy (Figure 9D), and TJP3 high-expression group accompanies with higher proportion of anti-PD1 response (CR and PR) (Figure 9E).

Based on above the results, *in vitro* experiments are performed to show the relation between TJP3 and immune escape. As results show, down-regulation of TJP3 leads down-regulation of PD-L1 (a 30% decrease, *p* < 0.01) (Figure 9F, 9G), while over-expression of TJP3 (*p* < 0.001) makes up-regulation of PD-L1 (Figure 9F, 9H).

### Construction of ARGs-based prognosis prediction model of breast cancer

**Figure 10:**
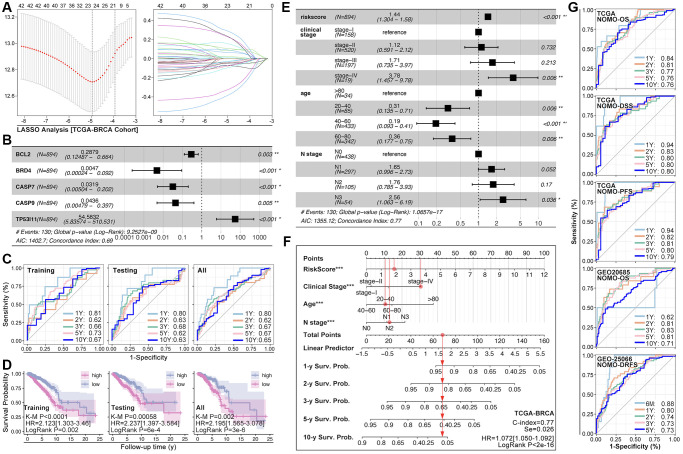
**ARGs-based nomogram.** (**A**) LASSO analysis and (**B**) Multivariate Cox regression screen out five ARGs (BCL2, BRD4, CASP7, CASP9, TP53I11) to construct prognosis prediction model. (**C**) ROC analysis of prognosis prediction model in training cohort, testing cohort, and all cohort (TCGA data). (**D**) K-M analysis of multi-gene riskscore model. (**E**) Multivariate Cox regression selects riskscore, clinical state, age and N stage as components to construct monogram (data from TCGA). (**F**) Visualization of monogram. (**G**) ROC analysis of monogram in TCGA cohort and GEO cohort.

**Figure 11:**
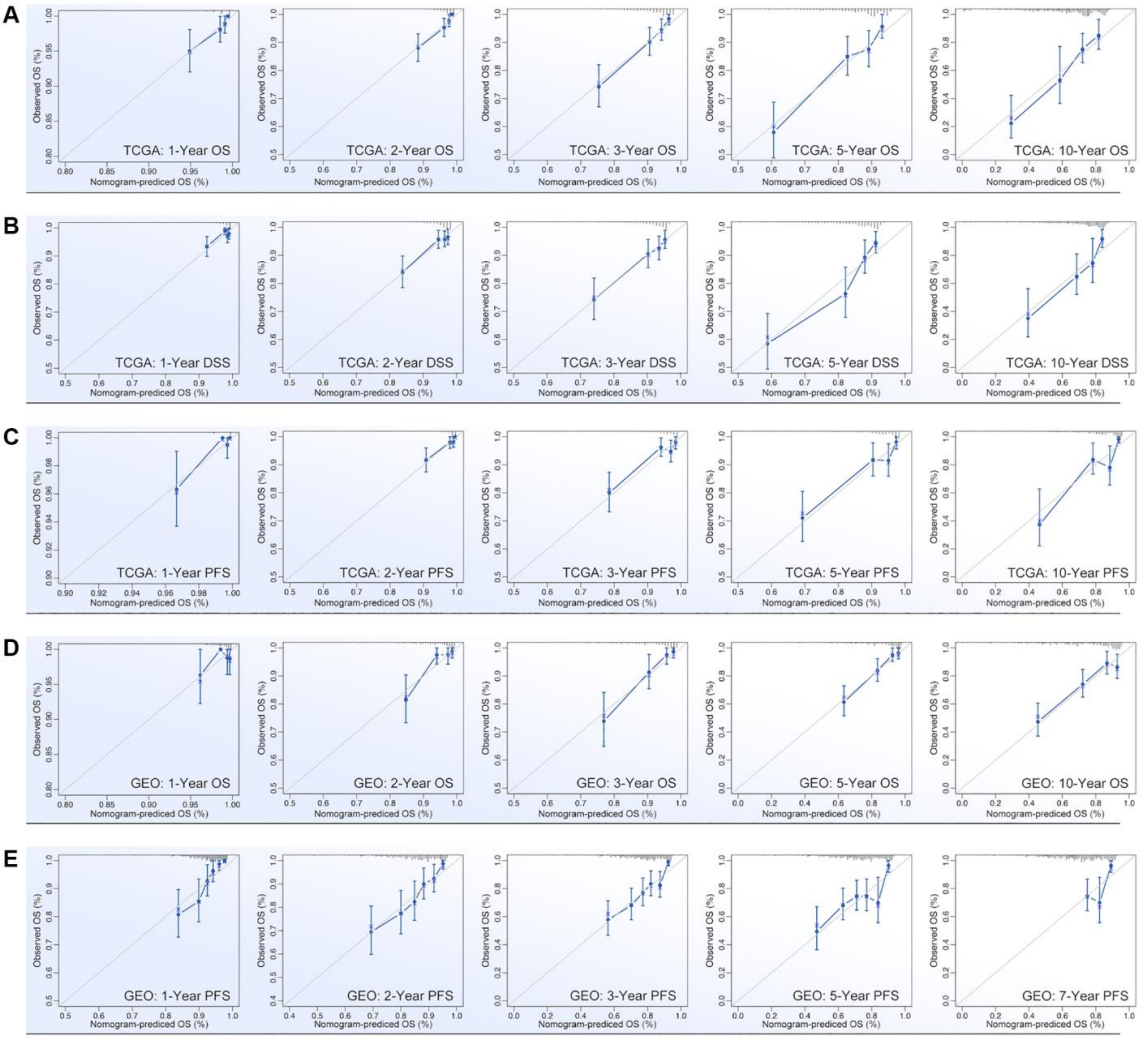
**Calibration of nomogram.** Calibration of (**A**) OS-prediction, (**B**) disease stable survival (DSS), (**C**) progression free survival (PFS) in monogram (data from TCGA). Calibration of (**D**) OS-prediction and (**E**) PFS in monogram (data from GEO).

ARGs show their ability in identifying drug sensitivity subgroups and prognosis differences, above. Here, prognosis prediction by ARGs is also explored. Firstly, LASSO analysis, Univariate Cox regression and Multivariate Cox regression are applied to screen out genes for constructing multi-gene risk model (TCGA cohort), in which BCL2, BRD4, CASP7 and TP53I11 are selected (Figure 10A, 10B). The concordance index of the model is 0.69 (*p* = 9.2527e-9, Figure 10B). Then, ROC and K-M analysis are performed, and the results show that only 1-year survival prediction AUC is greater than 0.8 (Figure 10C), and all of cohorts (training cohort, testing cohort, and whole cohort) show prognosis differences between high riskscore and low riskscore groups (Figure 10D). So, it’s necessary to add clinical features to improve the efficiency of risk model. As Figure 10E shows, Multivariate Cox regression selects riskscore, clinical stage, age and N stage to construct a nomogram, and the concordance index is 0.77 (*p* = 1.0657e-17, Figure 10E). Following, nomogram is visualized by R4.2.0, which is displayed in Figure 10F. To further verify the prediction efficiency of monogram, TCGA cohort and GEO cohorts are both put into ROC analysis. As the results show, the AUC value is obviously improved as compared with nomogram which without clinical features (Figure 10G). Besides, calibration analysis is also performed (Figure 11A–11E).

## DISCUSSION

Breast cancer develops to be a top1 incidence tumor type in human being now, which leads tumor-related death more than 600,000 per year [3]. Although the development of anti-HER2 therapy and novel antibody–drug conjugates (ADC) bring a large improvement of clinical outcome in breast cancer, about 30% of breast cancer turns into advanced stage, especially for TNBC [4]. Recently, immunotherapy showed an exciting progression in prolonging events-free survival (EFS) in early stage of TNBC (KENOTE-522 trial), which with positive expression of PD-L1 [16]. Thus, gene-detection-based individualized therapy holds a developing impact against advanced breast cancer.

Multi-gene-based risk model is reported to be applied in assessing prognosis and immunotherapy in various types of cancer. Recently, anoikis-related genes are considered as a tool to identify tumor immunity subtypes in liver cancer, lung cancer, and colorectal cancer [17–19]. In addition, anoikis is already reported to participate in breast cancer invasion before, such as HCXIP-mediated anoikis resistance leads increased ability of migration in breast cancer cells [20, 21]. Regretfully, the role of anoikis in drug sensitivity is still not clear yet now. A previous study shows that anoikis-resistant osteosarcoma is accompanied by enhanced drug tolerance (doxorubicin and cisplatin) [22]. In our study, we found ARGs are closely involved in drug resistance. As we are constructing ARGs-based AI model, we find AI identified ARG subgroups exist significant differences of drug score, in which ARG-1 subpopulation get highest drug score whereas ARG-3 subpopulation gets lowest drug score (Figure 6B, 6C). This implies patients with expression features like ARG-1 subpopulation probably have low response to chemotherapy, such as EPI, DTX, CTX, or PTX, et al. Besides, ARGs-based AI model successfully divides breast cancer into subgroups with prognostic differences, which is verified in independent cohorts in GEO data (Figure 5F, 5H, 5I). Based on pathway analysis, it shows that Hubgenes close to ARG subtype identification are involved into immunity process, such as interleukin-10 (IL10) and IL17 signaling pathways (Figure 4E, 4F), and are also involved into Wnt signaling pathway and Hippo signaling pathway, both of which participate in drug tolerance process in breast cancer [23–26].

TJP3 (tight junction protein 3), also named ZO-3, is considered as a cell scaffolding role which plays roles in epithelial differentiation [27]. Our study screens out TJP3 as a pivotal role in ARG subtype identification (Figure 7A). TJP3 is reported to participate in drug tolerance in long non-coding RNA NEAT1-mediaed tumor invasion in ovarian cancer [28], and it is involved in treatment sensitivity of FPDHP in human cancer cells [29]. In this study, we find up-regulation of TJP3 leads enhanced cell migration whereas down-regulation of it leads weaker migration ability and enhanced GEM-induced cell toxicity in TNBC cells (Figure 8A, 8D). Furthermore, down-regulation of TJP3 exactly reverses EMT, which performs as up-regulation of E-cad, down-regulation of MMP7, Twist1 and Zeb1 (Figure 9A, 9B). On contrary, up-regulation of TJP3 weakens GEM-induced cell apoptosis, such as cleaved caspase3 (Figure 9C, 9D). In addition, our research displays that TJP3 is overexpression in BRCA, CESC, COAD, OV, PAAD, READ, THYM, UCEC and UCS (Figure 7F).

## CONCLUSION

In this study, we explore the roles of anoikis-related genes in identifying tumor subgroups, assessing drug sensitivity and tumor immunity escape. Here, we consider that (1) ARGs-based AI model is useful to assess drug sensitivity in breast cancer; (2) ARGs-based monogram has potential merits for prognosis prediction in breast cancer; (3) An anoikis gene TJP3 promotes chemoresistance, cell migration and immunity escape in breast cancer by regulating EMT and PD-L1 expression. However, this paper doesn’t uncover the deeper investigation of the mechanisms of how TJP3 regulates PD-L1 and EMT process, it’s our further work.

## Supplementary Materials

Supplementary Figure 1
