# Supplementary material for: TJP3 promotes T cell immunity escape and chemoresistance in breast cancer: a comprehensive analysis of anoikis-based prognosis prediction and drug sensitivity stratification
Source: Aging (Albany NY). 2023 Nov 10;15(22):12890–906. doi: 10.18632/aging.205208 (PMC10713417; doi:10.18632/aging.205208)
Supplement: Supplementary Figure 1 [file aging-15-205208-s001.pdf]

SUPPLEMENTARY FIGURE

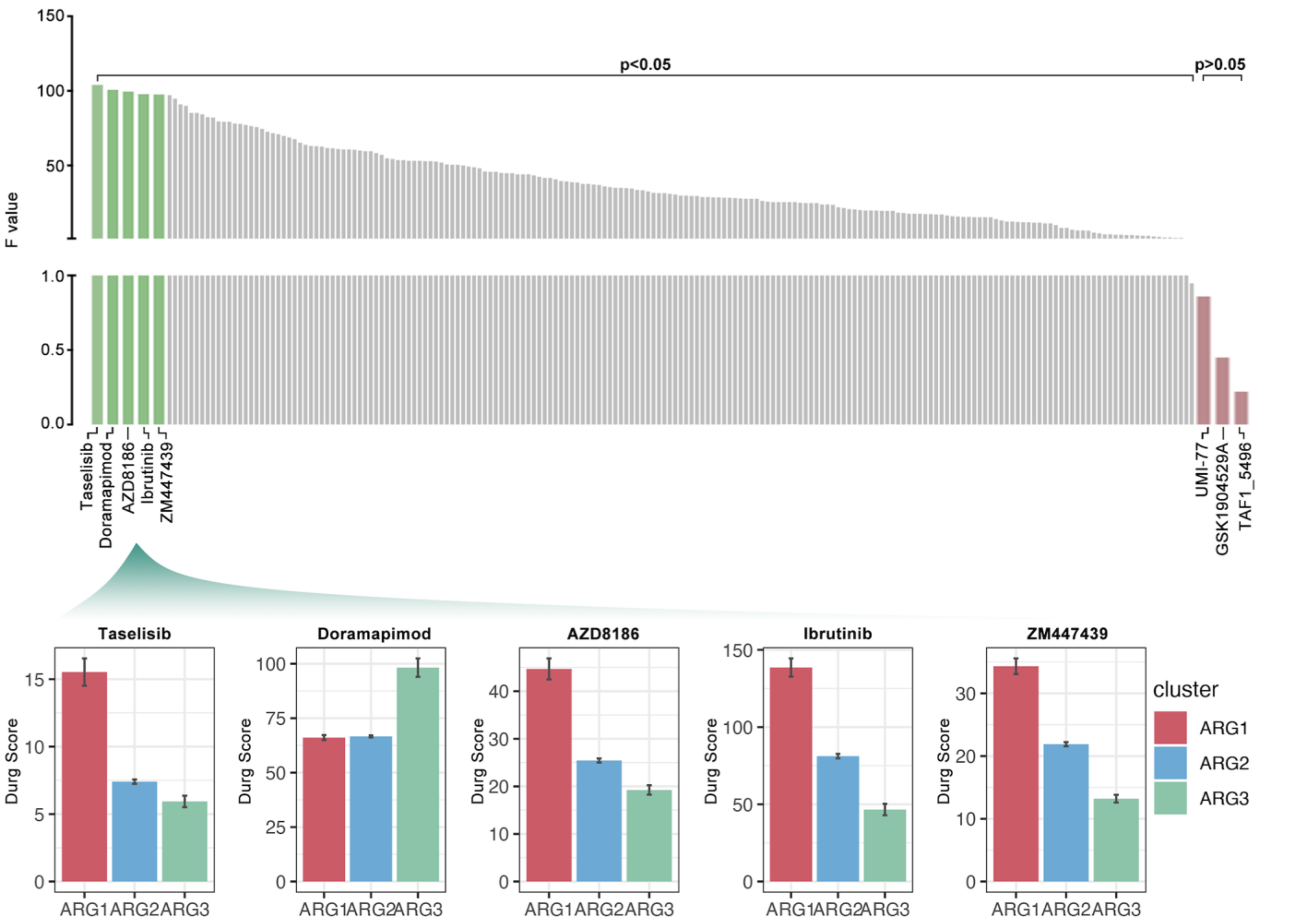

Supplementary Figure 1. Drug sensitivity. 199 kinds of drug are analyzed, 196 of which hold significant difference amongst ARG subgroup (tagged with green and grey). And Taselisib, Doramapimod, AZD8186, Ibrutinib and ZM447439 are top5 sensitive drugs, while UMI-77, GSK1904529A and TAF1 with no significant differences amongst ARG subgroups.
